# Proposing a highly accurate protein structural class predictor using segmentation-based features

**DOI:** 10.1186/1471-2164-15-S1-S2

**Published:** 2014-01-24

**Authors:** Abdollah Dehzangi, Kuldip Paliwal, James Lyons, Alok Sharma, Abdul Sattar

**Affiliations:** Institute for Integrated and Intelligent Systems (IIIS), Griffith University, Kessels Road, Brisbane, Australia; National ICT Australia (NICTA), Kessels Road, Brisbane, Australia; School of Engineering, Griffith University, Kessels Road, Brisbane, Australia; School of Engineering, The University of the South Pacific, Suva, Fiji Fiji

**Keywords:** Protein structural class prediction problem, Structural features, Evolutionary features, Segmented auto covariance, Segmented distribution, Support Vector Machine (SVM)

## Abstract

**Background:**

Prediction of the structural classes of proteins can provide important information about their functionalities as well as their major tertiary structures. It is also considered as an important step towards protein structure prediction problem. Despite all the efforts have been made so far, finding a fast and accurate computational approach to solve protein structural class prediction problem still remains a challenging problem in bioinformatics and computational biology.

**Results:**

In this study we propose segmented distribution and segmented auto covariance feature extraction methods to capture local and global discriminatory information from evolutionary profiles and predicted secondary structure of the proteins. By applying SVM to our extracted features, for the first time we enhance the protein structural class prediction accuracy to over 90% and 85% for two popular low-homology benchmarks that have been widely used in the literature. We report 92.2% and 86.3% prediction accuracies for 25PDB and 1189 benchmarks which are respectively up to 7.9% and 2.8% better than previously reported results for these two benchmarks.

**Conclusion:**

By proposing segmented distribution and segmented auto covariance feature extraction methods to capture local and global discriminatory information from evolutionary profiles and predicted secondary structure of the proteins, we are able to enhance the protein structural class prediction performance significantly.

**Electronic supplementary material:**

The online version of this article (doi:10.1186/1471-2164-15-S1-S2) contains supplementary material, which is available to authorized users.

## Background

Protein structural class prediction problem is defined as categorizing a given protein into one of the four structural classes namely, all-*α*, all-*β*, *α* + *β*, and *α*/*β* [1]. Knowledge of the structural classes of proteins can also provide important information about their functionalities and overall folding types [2, 3]. Therefore, protein structural class prediction problem is considered as an important step towards the protein structure prediction problem. Despite the importance of this problem, finding a fast and accurate computational approach to solve this problem when the sequence similarity rate is low still remains an unsolved problem for bioinformatics and computational biology.

During the past two decades, a wide range of studies, using machine learning-based methods, have been conducted to solve this problem [4, 5]. These studies can be categorized into two groups. The first group consists of studies that have tried to address this problem by proposing novel classification techniques [6, 7]. They proposed a wide range of classification techniques based on different learning algorithms such as, Bayesian based learners [8], *Meta-classifiers* [9–13], *Support Vector Machines (SVM)* [14–17], *Artificial Neural Network (ANN)* [18–20], and ensemble classifiers [21–25]. Among a wide range of classification techniques used to tackle this problem, SVM classifier has attained the best results for this task [5, 22, 26, 27]. The second group consists of studies that have mainly focused on proposing novel features that capture local and global discriminatory information to address protein structural class prediction problem such as sequence based information [10, 28–30], pseudo amino acid composition [31–33], physicochemical-based information [15, 22, 28, 34–36], and structural based information [5, 33, 37–40]. The most important enhancements in protein structural class prediction accuracy have been based on relying on these techniques rather than exploring the impact of classification techniques. These recent enhancements were mainly because of extracting features from *Position Specific Scoring Matrix (PSSM)* profiles [41] as well as structural information extracted from the predicted secondary structure of proteins [42].

The most significant enhancement by solely relying on the PSSM for feature extraction was achieved by [16, 26, 40]. They used PSSM profiles to extract sequence order information based on the concepts of dipeptide composition, auto covariance and composition of the amino acids. They used entire protein sequence as a general entity to extract these features. Hence, the auto covariance and dipeptide composition calculated along an entire protein sequence were used as its local descriptor. Further enhancement for protein structural class prediction accuracy has been achieved by including structural information extracted from the predicted secondary structure of the proteins using PSIPRED [42]. By adding these features to the extracted features from the PSSM, the protein structural class prediction accuracy has been significantly improved especially when the sequence similarity rate was low [27, 37, 43]. Similar to the features extracted from the PSSM, the whole protein as a general entity was used to extract these features as well. Despite all the recent efforts on extracting effective features to capture local and global discriminatory information from evolutionary and structural profiles, the protein structural class prediction accuracy have not been improved significantly since the study of Mizianty and Kurgan in 2009 [5, 6].

In this study, we propose segmented auto covariance and segmented distribution feature extraction methods to capture more local sequence order information from evolutionary and structural profiles. We also employe the concept of occurrence and composition feature groups to capture global sequence order information based on evolutionary, and structural profiles. First, by solely relying on the PSSM profiles for feature extraction, we enhance the protein structural class prediction accuracy by over 15% and 5% for 25PDB and 1189 benchmarks respectively compared to similar studies [26]. These enhancements highlight the potential discriminatory information embedded in the PSSM that have not been adequately explored in the literature. Then, by exploring our proposed feature extraction techniques to include structural information derived from the predicted secondary structure using SPINE-X [44], we achieve up to 92.2% and 86.3% prediction accuracies respectively for 25PDB and 1189 benchmarks and enhance the overall protein structural class prediction accuracy even further by 7.9% and 2.8% better than previously reported results found in the literature [5, 6, 27].

## Benchmarks

To evaluate the prediction performance of our proposed approaches, we employe two benchmarks namely 25PDB and 1189. These two benchmarks have been widely used for protein structural class prediction problem. The 25PDB was introduced by [45] consisting of 1673 proteins with less than 25% sequence similarities in average (the homology-range between 22% and 45%). This benchmark extracted from 25% PDBSELECTED which includes high-resolution non-homologous proteins from the *Protein Data Bank (PDB)* [46]. Therefore, it is considered as an appropriate representative of benchmarks consisting of proteins in twilight zone (proteins with sequence similarities between 20% and 45%) for protein structural class prediction problem. Hence, in this study, the 25PDB benchmark is used as the main source to investigate the effectiveness of our proposed model.

The other benchmark employed in this study is known as the 1189 benchmark. The 1189 benchmark was introduced by [8] consisting of 1189 proteins with less than 40% sequence similarities. This benchmark was modified in later studies to address further corrections of *Structural Classification of Proteins (SCOP)* [47] and 97 of its proteins were removed [45]. Therefore, later version of this benchmark consists of 1092 proteins. Sequences in this benchmark have lower resolution than proteins in the 25PDB benchmark. Therefore, despite higher sequence similarity in average among proteins in this benchmark compared to 25PDB benchmark, similar (or in many cases, even lower) protein structural class prediction accuracies has been reported for 1189 benchmark compared to 25PDB benchmark [5, 6, 24, 48]. Since, this benchmark has been widely used to investigate the performance of the methods used for protein structural class prediction problem, it is also adopted here to compare our achieved results directly with previously reported results found in the literature [45]. Employed benchmarks in this study and the number of proteins belonging to each structural class are shown in Table 1.

**Table 1:** The properties of 1189 and 25PDB benchmarks.

Benchmarks	All-***α***	All-***β***	***α/β***	***α + β***	Total
1189	223	294	334	241	1092
25PDB	443	443	346	441	1673

## Feature extraction methods

In this study, we use PSSM profiles to extract evolutionary-based information as well as predicted secondary structure using SPINE-X to extract structural-based information. PSSM is calculated by applying the PSI-BLAST [41] in which its cut off value (E) is set to 0.001 on our explored benchmarks (using NCBI's non redundant (NR) protein data base). Given a protein sequence, PSSM produces the substitution probability of the amino acids along its sequence based on their position with all 20 amino acids. PSSM consists of two *L ×* 20 matrices (*L* is the length of a protein and the columns of the matrices represent 20 amino acids). The first matrix is called PSSM_cons and gives the log-odd of the substitution probability. The second matrix is called PSSM_prob and gives the normalized substitution probability for each amino acid [27].

We also use predicted secondary structure using SPINE-X which was recently proposed by [44] and attained better results than PSIPRED on predicting protein secondary structure (especially for the coded area). Given a protein sequence, SPINE-X produces a *L ×* 3 matrix (which will be referred to SPINE-M for the rest of this study) including the normalized probability of contribution of a given amino acid based on its position along the protein sequence to build one of the three secondary structure elements namely, *α*-helix, *β*-strands, and coils. It also return a transformed version of the protein sequence (also extracted from the SPINE-M) in which each amino acid along the protein sequence is replaced with *H* (represents helix), *E* (represents strand), or *C* (represents coil) based on its tendency to incorporate in building one of these secondary structure elements. We will refer to this sequence as the structural consensus sequence. It is expected that predicted secondary structure using SPINE-X provides significant structural information for the protein structural class prediction problem similar to or even better than PSIPRED due to its better performance [44].

### Consensus sequence-based occurrence

To provide global discriminatory information about the sequence order of the amino acids along a protein sequence, we first extract the occurrence of the amino acids from the evolutionary consensus sequence as well as occurrence of secondary structure elements from the structural consensus sequence. As it was mentioned earlier, the structural consensus sequence is produced as one of the output of SPINE-X. The evolutionary consensus sequence is calculated based on the PSSM as follows. To extract this sequence, we replace a given amino acid along the original protein sequence (*O*_1_, *O*_2_, ..., *O*_*L*_) with an amino acid with maximum substitution probability in the row corresponding to the location of that amino acid in the PSSM (*CP*_1_*, CP*_2_*, ..., CP*_*L*_). This is done using the following two steps. In the first step, the index is found as:1

where *P*_*ij*_ is the substitution probability of the amino acid at location *i* with the *j-th* amino acid in the PSSM_cons. In the second step, we replace the amino acid at *i-th* location of original protein sequence by the *j-th* amino acid to form the consensus sequence. Note that the PSSM_cons is used in this study for feature extraction (which it is normalized using min-max method) as it was used in the literature [26, 27].

After calculating evolutionary consensus sequence, we count the occurrence of each amino acid (for all 20 amino acids) along this sequence and produce corresponding feature group (*AAO*). Similarly, we calculate the occurrence of each secondary structure element (for all three elements) in the structural consensus sequence and produce the corresponding feature group (*SSEO*). Occurrence feature group as the global descriptor of the proteins is used in this study instead of composition of the amino acids (occurrence of amino acids divided by the length of protein sequence) since it maintains the length information which is disregarded in the composition feature group [15].

### Semi-composition

In this method, we calculate semi-composition feature group from both PSSM and SPINE-M. It is called semi-composition because instead of using the protein sequence directly to calculate the composition of each amino acid along the protein sequence (as it was done conventionally [27]), we calculate the summation of the substitution probability for each amino acid directly from the PSSM (similar to [26]) or normalized frequency of each secondary structure element from the SPINE-M. The semi-composition derived from the PSSM (*PSSM-AAC*) is calculated as follows:2

In the similar manner, we calculate the semi-composition of each secondary structure element by adding the normalized frequencies of the corresponding element from the SPINE-M (*SPINE-SSEC*) as follows:3

where *S*_*ij*_ is the normalized probability of the occurrence of the *j-th* secondary structure element at location *i* of the protein sequence in the SPINE-M. It was shown that using semi-composition method is able to provide more discriminatory information compared to extracting composition of the amino acids feature group from the original protein sequence [26]. This feature group is also able to provide important global discriminatory information about the substitution probability of the amino acids as well as normalized frequency of secondary structure elements.

### Segmented distribution

This method is specifically proposed to add more local sequence order information about how the amino acids based on their substitution probability with each other (extracted from the PSSM) as well as their tendency to incorporate in one of the secondary structure elements (extracted from SPINE-M) are distributed along the protein sequence. We propose this segmentation method in the manner where segments of a protein sequence are of unequal lengths and each segment is represented by a distribution feature which is computed as follows. First, for the PSSM, to extract the segmented distribution feature group (*PSSM-SD*), we compute the total sum of substitution probability of the *j* column of the PSSM . Then, we start from the first row of the PSSM and compute the partial sum of the substitution probability of the amino acid amino acid *j*, for the first *i* amino acids which is given by . Using the distribution factor *F*_*P*_ (which is a parameter investigated in this study), we find out the maximum value  of index *i* such that partial sum *S*_1_ is less than or equal to the *F*_*P*_% of total sum (*T*_*j*_). Thus we can say that the first ?6? substitution probabilities contribute to *F*_*P*_% of the total sum (*T*_*j*_). We use ?6? to define the ending location of the first segment, while its beginning point is taken to be 1 (which represents the first row of the PSSM). The distribution feature of this segment is given by ?6?. In a similar manner, we find out the number of first  amino acids of the protein sequence that contribute to 2*F*_*P*_%, 3*F*_*P*_%, ..., 50% of *T*_*j*_ (50% of *T*_*j*_ starting from the first row of the PSSM), respectively. Indices , are used to define the ending locations of segments 2, 3, ..., 50/*F*_*P*_ , respectively; while the beginning location of all these segments remains to be 1. Hence, the distribution features for these segments are computed as . Note that we have thus computed 50/*F*_*P*_ distribution features by processing the protein sequence starting from the first row of the PSSM in downward direction. We repeat this process starting from the last row of the PSSM in upwards direction to get another set of 50/*F*_*P*_ features (to explore the rest of 50% of *T*_*j*_ starting from the end of protein sequence corresponding to the last row of the PSSM). Thus, the total of 2× (50/*F*_*P*_) = 100/*F*_*P*_ distribution features are computed for each column of the PSSM.

**Figure 1:**
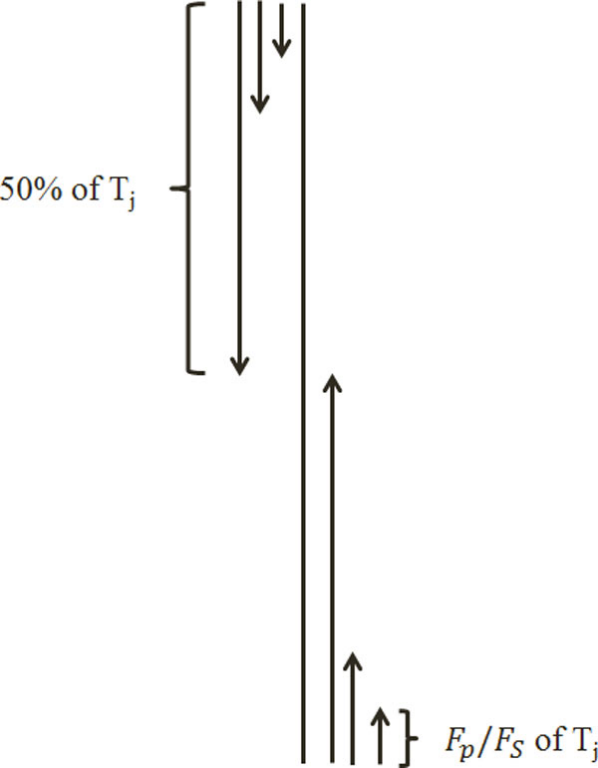
**Feature extraction scheme using the segmented distribution method**.

The distribution factor (*F*_*P*_) is a parameter which is determined here experimentally. For this, three values of *F*_*P*_ (5, 10, and 25) are investigated. Thus there will be 20, 10, and 4 features for *F*_*P*_ = 5, 10 and 25, respectively for the *j*-th column of the PSSM. Since there are 20 amino acids (corresponding to 20 columns in the PSSM) we produce 20 × 20, 20 × 10, and 20 × 4 features corresponding to *F*_*P*_ = 5, 10, and 25, respectively. In the similar manner, we calculate the segmented distribution of the normalized frequency of the secondary structure elements from the SPINE-M (*SPINE-SD*) using *F*_*S*_ = 5, 10, and 25 (where *F*_*s*_ is used as the distribution factor for the SPINE-M equivalent to *F*_*P*_ used for the PSSM) and extract 3 × 20, 3 × 10, and 3 × 4 features in total for all three elements, respectively. This procedure is shown in Figure 1.

### Segmented auto covariance

The concept of auto covariance have been widely used in the literature to capture local sequence order information and attained better results compared to similar methods used for this task such as dipeptide composition [15, 48, 26, 49]. Pseudo amino acid composition based features are good examples of these types of features [4, 50]. These features have been computed using the whole protein sequence as a single entity for feature extraction. Therefore, they are not able to adequately explore the local sequence order information embedded in the protein sequence [26]. In the present study, we extend the concept of segmented distribution features as described in the previous subsection to compute the auto covariance features from the segmented protein sequence. This is done to provide more evolutionary and structural sequence order information both from the PSSM and SPINE-M. First for the PSSM, we segment the protein sequence using distribution factor of 25% (*F*_*P*_ = 25) until reaching to *F*_*P*_ = 50 from each side (for the *j-th* column). Using a procedure similar to the one described in the previous subsection in which *F*_*P*_ = 25, we calculate . These indices are used to divide protein sequence into four segments as follows: From the first amino acid (corresponding to the first row of the PSSM) to ?6?; From the first amino acid (corresponding to the first row of the PSSM) to ; From the last amino acid (corresponding to the last row of the PSSM) to ; And from the last amino acid (corresponding to the last row of the PSSM) to . Then we calculate *K*_*P*_ (distance factor used for the PSSM) numbers of auto covariance coefficients for each of these segments as follows:4

where, *P*_*ave, j*_ is the average substitution probability for the *j-th* column in the PSSM (for 20 columns). Note that 4 × *K*_*P*_ auto covariance coefficients are computed in this manner (2 × *K*_*P*_ features by analyzing the PSSM in the downward direction and 2 × *K*_*P*_ features by analyzing the PSSM in the upward direction). We also compute the global auto covariance coefficient (*K*_*P*_ features) corresponding to the *j-th* column to provide more information as follows:5

Thus, we have extracted a total of (2*K*_*P*_ + 2*K*_*P*_ + *K*_*P*_ = 5*K*_*P*_) auto covariance features in this manner (PSSM-seg + PSSM-AC). Therefore, for PSSM, for all of the amino acids (all 20 columns of the PSSM) segmented auto covariance of substitution probability of the amino acids are extracted and combined to build the corresponding feature group ( PSSM-SAC which consists of 20 × (2*K*_*P*_ + 2*K*_*P*_ + *K*_*P*_) features in total). This procedure is also repeated for SPINE-M in the similar manner (where *K*_*S*_ is adopted as the distance factor for the SPINE-M equivalent to *K*_*P*_ used for the PSSM). For all three secondary structure elements we calculate segmented auto covariance of normalized frequency of secondary structure elements as follows:6

where, *S*_*ave, j*_ is the average substitution probability for the *j-th* column in the SPINE-M. Similarly, the global auto covariance corresponding to the *j-th* column in SPINE-M is computed and added to this feature group as follows:7

Combining SPINE-seg and SPINE-AC, we build SPINE-SAC feature group consisting of 3 × (2*K*_*S*_ + 2*K*_*S*_ + *K*_*S*_)) features in total (4*K*_*S*_ features in SPINE-seg and *K*_*S*_features in SPINE-AC).

## Support Vector Machine (SVM)

SVM was introduced by [51] aiming to find the *Maximal Margin Hyperplane (MMH)* based on the concept of the support vector theory to minimize the error. It transforms the input data to higher dimension using the kernel trick to be able to find support vectors (for nonlinear cases). The classification of some known points in input space **x**_*i*_ is *y*_*i*_ which is defined to be either -1 or +1. If *x′* is a point in input space with unknown classification then:8

where *y′* is the predicted class of point **x***′*. The function *K()* is the kernel function; *n* is the number of support vectors and *a*_*i*_ are adjustable weights and *b* is the bias. This classier is considered as the state-of-the-art classification techniques in the pattern recognition and attained the best results for the protein structural class prediction problem [5, 6, 26, 27]. In this study, SVM classifier implemented in the LIBSVM toolbox using Radial Base Function (RBF) as its kernel is used [52]. RBF kernel is adopted in our experiments due to its better performance than other kernels functions (e.g. polynomial kernel, linear kernel, and sigmoid [5, 6]). RBF kernel is defined as follows:9

where *γ* is the kernel parameter, **x**_**i**_ and **x**_**j**_ are input feature vectors. In this study, the *γ* in addition to the cost parameter *C* (which also called the soft margin parameter) of the SVM classifier are optimized using grid search algorithm implemented in the LIBSVM package. The grid search algorithm tries various pairs of *γ* and *C* values and selects the values with the best classification accuracy [52] (using 10-fold cross validation evaluation method). The range of gamma and C parameters to be searched in this algorithm are taken to be their default values used in the SVMLIB toolbox (these ranges were from 2^-5^ to 2^15^ for *C* and from 2^-15^ to 2^3^ for gamma). It is a simple algorithm as it has just two parameters to optimize (*γ* and *C*). Despite its simplicity, it has been shown to be an effective method to optimize these parameters [26].

## Results and discussion

We first investigate the effectiveness of our proposed feature extraction methods to capture local and global discriminatory information from the PSSM. We compare their performances with similar studies that relied solely on the PSSM for feature extraction [26]. In this step, we also explore the effective value for distance factor (*K*_*P*_) in segmented auto covariance feature extraction method as well as segmentation factor (*F*_*P*_) in segmented distribution method. To find the effective value for segmented auto covariance method, we study the *K*_*P*_ value between 1 and 10 (similar to [26]). We also study the segmentation factor (*F*_*P*_) in segmentation distribution between three values used in this study (25, 10 and 5). In the second step, we conduct a similar experiments using the SPINE-X for feature extraction. We investigate the effectiveness of our proposed feature extraction method to extract these features from the SPINE-M as well as the effective values for *K*_*S*_ (between 1 and 10) and *F*_*S*_ (among three values (25, 10, and 5) used in this study) in the similar manner. In the final step, we add the structural features extracted from the SPINE-M using our proposed methods to the extracted features from the PSSM and compare our results with the best results found in the literature for the protein structural class prediction problem [5, 6, 27].

To explore the impact of the distance factor on the segmented auto covariance method, 10-fold cross validation is adopted as it was widely used in similar studies [26, 45]. In this paper, we have used k-fold cross validation where *k* = 10 to measure the prediction performance. We also provide these performance results using k-fold cross validation as a function of *k* where *k* = 2, 3, 4, ..., 10 in Additional File 1. In the 10-fold cross validation, the benchmark is divided into ten non-overlapping subsets called fold. Then in each iteration, the combination of nine folds is used for training purpose and the remained fold is used for testing purpose. This process repeats for all 10 folds to be used as the testing set. We also use Jackknife cross validation to report our overall achieved prediction accuracy as well as prediction accuracy achieved for each structural class individually to compare them with previous studies. In this method, in each iteration, all but one sample use as a training purpose while the remained sample is used for testing purpose. This process repeats for all the samples available in the benchmark to be used as the testing sample. Jackknife is considered as a computationally expensive approach for evaluation. Furthermore, it was shown in [45] that its performance is similar to 10-fold cross validation. Since it has been widely used to evaluate protein structural class prediction accuracy, it is also adopted in this study to enable us to directly compare our results with the state of the art results found in the literature [5, 6, 26, 27]. We will use the overall prediction accuracy (in percentage) as the main accuracy measurement to be able to directly compare our achieved results with previously reported results found in the literature which is defined as follows:

10

where *C* is the number of correctly classified test samples and *N* is the total number of test samples. We will also report the *sensitivity*, *specificity* and *Matthews Correlation Coefficient (MCC)* measurements for each structural class to provide more information about the statistical significant of our achieved results [27, 45]. Sensitivity measures the proportion of correctly classified proteins compared to the whole number of samples which are classified as correct (correct versus incorrect) and is calculated as follows:11

where *TP* is the number of correctly identified (true positive) samples, while *FN* is the number of incorrectly rejected samples (false negative). On the other hand, specificity measures the proportion of the number of correctly rejected samples compared to the whole number of rejected samples (correctly versus incorrectly) and is calculated as follows:12

where *TN* is the number of correctly rejected (true negative) samples while *FP* is the number of incorrectly accepted samples (false positive). These two parameters are closely related to the prediction error and a predictor which is 100% sensitive and specific is considered as a perfect predictor (while 0% sensitive and specific is opposite). On the other hand, MCC measures the classification correlation and varies between -1 and 1 (where 1 indicates higher prediction quality while -1 indicate lower prediction quality and 0 indicate random correlation) and calculated as follows:13

More information about these three measurement for protein structural class prediction problem can be found in [27] and [45]. We will report sensitivity as well as specificity and MCC measures for all four structural classes for the best results reported in this study.

### Exploring the impact of our proposed methods relying only on PSSM for feature extraction

**Figure 2:**
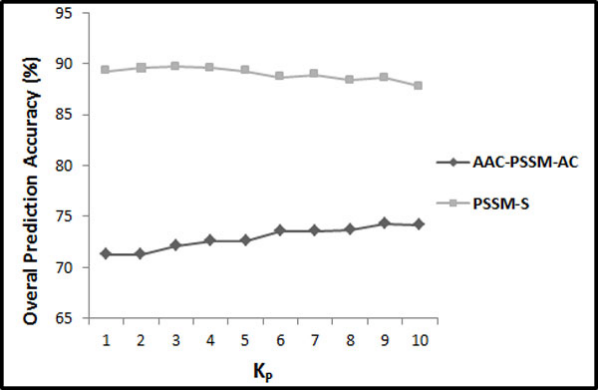
**The overall accuracies of PSSM-S compared to AAC-PSSM-AC for 25PDB benchmark**.

**Figure 3:**
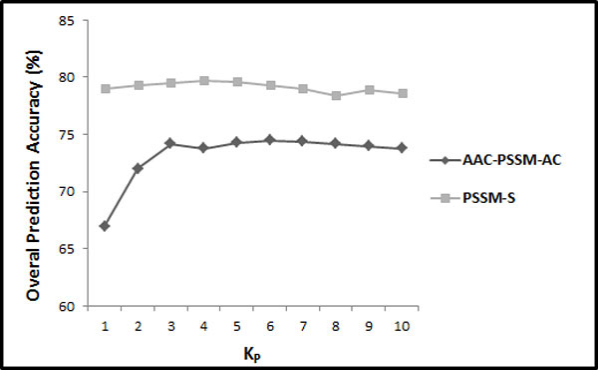
**The overall accuracies of PSSM-S compared to AAC-PSSM-AC for 1189 benchmark**.

In this step, we first extract the feature vector proposed by [26] and reproduce their results with respect to different distance factors (between 1 and 10). Their explored feature vector consists of semi-composition (PSSM-AAC) and global auto covariance (PSSM-AC) features extracted from the PSSM (called ACC-PSSM-AC). In continuation, we build a feature vector based on our proposed feature extraction methods in this study relying solely on the PSSM for feature extraction. We extract AAO (occurrence of the amino acids extracted from evolutionary consensus sequence (20 features)), PSSM-AAC (semi-composition from PSSM (20 features)), PSSM-SAC (segmented auto covariance in which *K*_*P*_ has been adjusted to 1 to 10 in 10 different experiments (*K*_*P*_ × 5 × 20 features)), and PSSM-SD (segmented distribution in which segmentation factor has been adjusted to 25 (4 × 20 = 80 features)) feature groups. The combination of these feature groups is referred as PSSM-S (AAO + PSSM-AAC + PSSM-SD + PSSM-SAC = PSSM-S). The results achieved by reproducing [26] experiment compared to our results with respect to different values of *K*_*P*_ (between 1 and 10) for the 25PDB and 1189 benchmarks are shown in Figure 2 and Figure 3 respectively.

Note that we optimized *γ* and C for *K*_*P*_ = 1 and *F*_*P*_ = 25 using grid algorithms on the 1189 benchmarks (to avoid over tuning) and used corresponding values for the rest of this study (*γ* = 0.055 and *C* = 500). We determine the parameters used in this study for feature extraction as well as employed classification technique on the 1189 benchmark while the 25PDB is not used at all and reserved to investigate the generality and effectiveness of our proposed model. However, our experiments have determined that there is no significant difference between the optimized parameters for the 25PDB and 1189 benchmarks for our extracted features.

As we can see in Figure 2 and Figure 3, our extracted feature vector significantly outperforms the results reported in [26] for all the values used for *K*_*P*_ (between 1 and 10). It shows the effectiveness of the proposed segmentation-based method to explore discriminatory information embedded in the PSSM compared to use of whole protein sequence as a general entity. It also shows that by using segmented auto co-variance method, even by using very low values for *K*_*P*_, we can achieve to high prediction accuracy since it is able to explore adequate local sequence order information (also emphasis on the impact of segmented distribution method). We report up to 89.6% prediction accuracy (using jackknife cross validation) by adjusting *K*_*P*_ to 4 (20 + 20 + 5 × *K*_*P*_ (= 4) × 20 + 80 = 520 features in total) which is 15.5% better than 74.1% prediction accuracy achieved by reproducing [26] experiment (using *K*_*P*_ = 9 in AAC_PSSM_AC) for the 25PDB benchmark (Figure 2). Similarly, we achieve up to 79.7% prediction accuracy by adjusting *K*_*P*_ to 4 which is 5.1% better than 74.6% prediction accuracy achieved by reproducing [26] experiment (using *K*_*P*_ = 6 in AAC_PSSM_AC) for the 1189 benchmark (Figure 3). Since the best results for both 25PDB and 1189 benchmarks are achieved by setting *K*_*P*_ to 4 (the achieved results do not differ significantly for different values used for *K*_*P*_ (between 1 and 10) which highlights the effectiveness of segmentation technique rather than the effect of the distance factor (*K*_*P*_) to extract this feature group), it is adopted as a distance factor to extract features for segmented auto covariance from the PSSM for the rest of this study.

We also repeat this experiment to explore the impact of segmentation factor *F*_*P*_ in segmented distribution feature extraction method. The prediction accuracies achieve by adjusting the segmentation factor to 10 and 5 are not improved (which even by increasing *K*_*P*_, they are reduced) compared to the achieved results by adjusting this parameter to 25. It highlights the sufficiency and effectiveness of adopting *F*_*P*_ = 25 as the segmentation factor compare to use of 10 and 5. In other word, using four segments is able to effectively provide adequate discriminatory information for this task better than increasing the number of segments to 10 or 20.

In Table 2, we show the prediction accuracy achieved by adding proposed feature groups (in which *K*_*P*_ = 4 and *F*_*P*_ = 25) in this study one by one to PSSM-AAC to build PSSM-S (for both 25PDB and 1189 benchmarks). In this manner, we can investigate the effectiveness of each feature group individually on the reported prediction accuracy. As we can see, adding PSSM-SAC and PSSM-SD significantly enhance the protein structural class prediction accuracy which highlights the impact of segmentation approach to provide significant discriminatory information for this task.

**Table 2:** The impact of the proposed feature extraction groups (using PSSM for feature extraction) proposed in this study to enhance protein structural class prediction accuracy (in %).

Combination of features	25DDB	1189
PSSM-AAC	64.3	61.2
PSSM-AAC + PSSM-SAC	69.4	68.0
PSSM-AAC + PSSM-SAC + PSSM-SD	88.6	77.9
PSSM-AAC + PSSM-SAC + PSSM-SD + AAO	89.6	79.7

### Exploring the impact of our proposed methods relying only on SPINE-X for feature extraction

In this step, we investigate the impact of our proposed feature extraction method on the SPINE-X for feature extraction. We build a feature vector based on our proposed methods in this study relying solely on the SPINE-M for feature extraction. We extract SSEO (occurrence of the secondary structure elements from predicted secondary structure using SPINE-M (3 features)), SPINE-SSEC (semi-composition from SPINE-M (3 features)), SPINE-SAC (segmented auto covariance were *K*_*S*_ adjust to 1 to 10 in 10 different experiments (*K*_*S*_ × 5 × 3 features)), and SPINE-SD (segmented distribution where segmentation factor adjusts to 25 (4 × 3 = 12 features)) feature groups. The combination of these feature groups is referred as SPINE-S (SSEO + SPINE-SSEC + SPINE-SD + SPINE-SAC = SPINE-S). The protein structural class prediction results are obtained in this subsection using the Jack-knife cross validation method.

**Figure 4:**
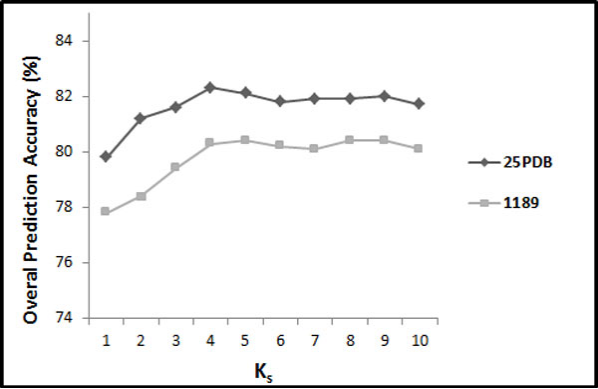
**The overall accuracies of SPINE-S with respect to different values of** ***K***_***S***_ **for 25PDB and 1189 benchmarks (where** ***F***_***S***_ **= 25)**.

The results achieved for SPINE-S with respect to different values of *K*_*S*_ (between 1 and 10) for the 25PDB and the 1189 benchmarks are shown in Figure 4. These results are obtained with distribution factor *F*_*S*_ = 25. As we can see in Figure 4, these SPINE-S features give best results for *K*_*S*_*≥* 4. For *K*_*S*_ = 4, these features produce 82.3% for the 25PDB benchmark and 80.3% for the 1189 benchmark. Note that these results are comparable to their corresponding PSSM results reported in Section 5.1. This shows the effectiveness of the proposed segmentation-based method to explore discriminatory information from the SPINE-M (similar to the PSSM). For *K*_*S*_ = 4, the feature vector has 78 features (3 + 3 + 5 × *K*_*S*_ (= 4) × 3 + 12 = 78). Furthermore, we have studied the SPINE-S features for distribution factor (*F*_*S*_) having values 5, 10, and 25. We have found that all the three values of *F*_*S*_ gave similar results. Therefore, we have reported the results for *F*_*S*_ = 25.

In Table 3, we show the prediction accuracy achieved by adding proposed feature groups (in which *K*_*S*_ = 4 and *F*_*S*_ = 25) in this study one by one to SPINE-SSEC to build SPINE-S (for both of the 25PDB and 1189 benchmarks). In this manner, we can investigate the effectiveness of each feature group individually on the reported prediction accuracy. We can observe from Table 3 that addition of SPINE-SAC and SPINE-SD has enhanced the protein structural class prediction accuracy, similar to PSSM.

**Table 3:** The impact of the proposed feature extraction groups (using SPINE-M for feature extraction)proposed in this study to enhance protein structural class prediction accuracy (in %).

Combination of features	25DDB	1189
SPINE-AAC	78.2	75.1
SPINE-AAC + SPINE-SAC	79.2	78.2
SPINE-AAC + SPINE-SAC + SPINE-SD	81.6	79.0
SPINE-AAC + SPINE-SAC + SPINE-SD + SSEO	82.3	80.3

### Exploring the impact of our proposed method using both PSSM and SPINE-X for feature extraction

**Figure 5:**
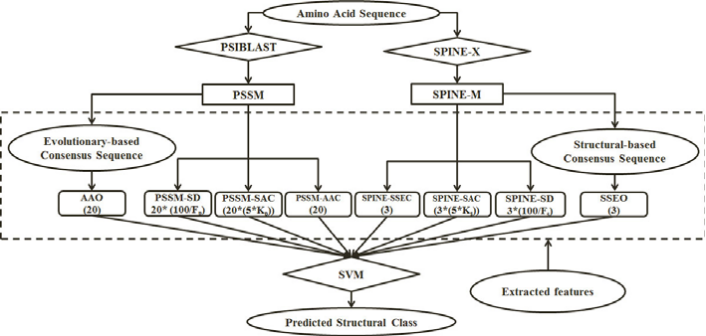
**The general architecture of our proposed feature extraction model**. The number of features extracted in each feature group is shown in the brackets below the feature groups' names.

In continuation we investigate the effectiveness of our proposed feature extraction methods to extract structural information from the SPINE-X and add these features to evolutionary information extracted from the PSSM. We extract SSEO (3 features), SPINE-SSEC (3 features), SPINE-SAC (where *K*_*S*_ adjusted from 1 to 10 in 10 different experiments (*K*_*P*_*×* 5 × 3 features)), and SPINE-SD (where *F*_*S*_ = 25 for the SPINE-M). The general architecture of our proposed feature extraction model is shown in Figure 5. The combination of the extracted features from the PSSM and the SPINE-M is referred to as PSSM-SPINE-S for the rest of this study (AAO + PSSM-AAC + PSSM-SAC + PSSM-SD + SSEO + SPINE-AAC + SPINE-SAC + SPINE-SD = PSSM-SPINE-S).

**Figure 6:**
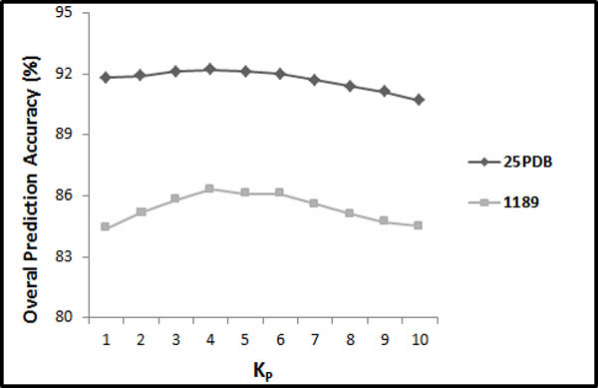
**The overall accuracies of PSSM-SPINE-S with respect to different values of** ***K***_***S***_ **for 25PDB and 1189 benchmarks (where** ***K***_***P***_ **= 4 and** ***F***_***P***_ **= 25%)**.

In the first step, we set the segmentation factor (*F*_*S*_) to 25 and adjust distance factor (*K*_*S*_) between 1 and 10 and add these features to the extracted features from the PSSM (while for the PSSM, distance factor is set to *K*_*P*_ = 4 and segmentation factor is set to 25 which is investigated earlier in Section 5.1). We conduct 10 experiments by adjusting *K*_*S*_ from 1 to 10 in this step (using jackknife cross validation). The results achieved for both of the 25PDB and 1189 are shown in Figure 6. In this part, for the first time we enhance the protein structural class prediction accuracy to over 90% for 25PDB benchmark and 85% for 1189 benchmark. By adjusting *K*_*S*_ = 4 (similar to the distance factor adopted to extract segmented auto covariance feature group from the PSSM) and segmentation factor *F*_*S*_ = 25 (similar to *F*_*P*_) we achieve up to 92.2% and 86.3% prediction accuracies for both of the 25PDB and 1189 benchmarks (20 + 20 + 5 × *K*_*P*_ (= 4) × 20 + 80 + 3 + 3 + 5 × *K*_*S*_ (= 4) × 3 + 12 = 598 features in total), up to 7.9% and 2.8% better than previously reported results for these two benchmarks using evolutionary and structural features simultaneously [6, 27, 5].

These enhancements achieved by increasing the prediction accuracy for all of the structural classes monotonically. We achieve to over 90% prediction accuracies (sensitivity) for three structural classes for the 25PDB benchmark (96.8%, 93.7%, and 90.1% prediction accuracies for all-*α*, all-*β*, and *α/β* structural classes, respectively). We also report 87.0% prediction accuracy for *α* + *β* structural class, which is considered as a difficult structural class to predict which is 9.4% over the highest results reported for this structural class [48]. Despite the results achieved for the 1189 benchmark have not been as high as the results achieved for the 25PDB benchmark, they still have been significantly better than the reported results for this benchmark (especially by considering that it has not been improved since the study of Mizianty and Kurgan in 2009). We also report 98.2%, 91.5%, and 72.2% prediction accuracies for all-*α*, all-*β*, and *α* + *β* structural classes which are respectively 4.5%, 4.1% and 1.2% over the best results reported for these structural classes in the literature [6, 5]). The results achieved (overall prediction accuracy as well as sensitivity for each structural class) in this study compared to previously reported results for the 25PDB and 1189 benchmarks are shown in Table 4 and Table 5, respectively.

**Table 4:** Comparison of the results reported for the 25PDB benchmark (in percentage %)

References	Method	All-***α***	All-***β***	***α***/***β***	***α*** + ***β***	Overall
[45]	Logistic Regression	69.1	61.6	60.1	38.3	57.1
[53]	Specific Tri-peptides	60.6	60.7	67.9	44.3	58.6
[33]	LLSC-PRED	75.2	67.5	62.1	44.0	62.2
[33]	SVM	77.4	66.4	61.3	45.4	62.7
[38]	AAD-CGR	64.3	65.0	65.0	61.7	64.0
[7]	CWT-PCA-SVM	76.5	67.3	66.8	45.8	64.0
[54]	AATP	81.9	74.7	75.1	55.8	71.7
[16]	AADP-PSSM	83.3	78.1	76.3	54.4	72.9
[55]	SCPRED	92.6	80.1	74.0	71.0	79.7
[37]	SSA	92.6	83.7	80.5	65.9	81.5
[37]	PSSA	94.6	76.3	73.1	74.4	80.0
[24]	RKS-PPSC	92.8	83.3	80.8	70.1	82.9
[48]	SVM	92.6	81.3	81.5	76.0	82.9
[27]	MODAS	92.3	83.7	81.2	68.3	81.4
[26]	AAC-PSSM-AC	85.3	81.7	73.7	55.3	74.1
[22]	Physicochemical-based features	86.1	80.8	80.6	60.1	76.7
[5]	Structural-based features	95.0	85.6	81.5	73.2	83.9
[6]	Structural-based features	95.0	81.3	83.2	77.6	84.3
This Study	PSSM-S	93.5	90.3	92.1	81.4	**89.6**
This Study	SPINE-S	93.8	83.1	78.4	73.9	**82.3**
This Study	PSSM-SPINE-S	**96.8**	**93.7**	**90.1**	**87.0**	**92.2**

**Table 5:** Comparison of the results reported for the 1189 benchmark (in percentage %)

References	Method	All-***α***	All-***β***	***α***/***β***	***α*** + ***β***	Overall
[8]	Bayes Classifier	54.8	57.1	75.2	22.2	53.8
[45]	Logistic Regression	57.0	62.9	64.7	25.3	53.9
[56]	FKNN	48.9	59.5	81.7	26.6	56.9
[57]	WSVM	-	-	-	-	59.2
[53]	Specific Tri-peptides	-	-	-	-	59.9
[21]	IB1	65.3	67.7	79.9	40.7	64.7
[38]	AAD-CGR	62.3	67.7	66.5	63.1	65.2
[58]	SVM	75.8	75.2	82.6	31.8	67.6
[54]	AATP	72.7	85.4	82.9	42.7	72.6
[16]	AADP-PSSM	69.1	83.7	85.6	35.7	70.7
[55]	SCPRED	89.1	86.7	89.6	53.8	80.6
[24]	RKS-PPSC	89.2	86.7	82.6	65.6	81.3
[27]	MODAS	92.3	87.1	87.9	65.4	83.5
[26]	AAC-PSSM-AC	80.7	86.4	81.4	45.2	74.6
[22]	Physicochemical-based features	80.2	83.6	85.4	44.6	74.8
[5]	Structural-based features	92.4	87.4	82.0	71.0	83.2
[6]	Structural-based features	93.7	84.0	83.5	66.4	82.0
This Study	PSSM-S	92.6	86.0	76.7	64.3	**79.7**
This Study	SPINE-S	91.9	88.3	78.9	61.7	**80.3**
This Study	PSSM-SPINE-S	**98.2**	**91.5**	**83.8**	**72.2**	**86.3**

Adding structural features to evolutionary features extracted in our experiments enhances the results for up to 2.4% and 6.6% better than relying solely on evolutionary features for the 25PDB and 1189 benchmarks respectively. This emphasis on the impact of structural information extracted from the SPINE-X in general for the protein structural class prediction problem.

We also provide the specificity and MCC for the best results reported in this study (results achieved for the PSSM-S, SPINE-S, and PSSM-SPINE-S) for the 25PDB and 1189 benchmarks in Table 6. As we can see, high values for specificity (near 100%) similar to the high sensitivity values in Table 4 and Table 5 (near 100%) as well as MCC values (which are all higher than 0.5) for our achieved results support the statistical significant of our reported results in this study.

**Table 6:** The specificity (in percentage) and MCC measurements for the best results: (a) for the 25PDB benchmark; (b) for the 1189 benchmark

Feature Vector	Specificity (%)				MCC			
	**All-** ***α***	**All-** ***β***	***α*** **/** ***β***	***α*** **+** ***β***	**All-** ***α***	**All-** ***β***	***α*** **/** ***β***	***α*** **+** ***β***
(a) PSSM-S	97.7	96.3	95.2	91.9	0.93	0.80	0.78	0.91
SPINE-S	97.8	94.0	94.4	90.5	0.89	0.80	0.75	0.61
PSSM-SPINE-S	98.9	97.7	96.7	96.4	0.94	0.89	0.86	0.87
(b) PSSM-S	98.2	94.8	89.8	90.0	0.91	0.78	0.67	0.56
SPINE-S	97.9	95.8	90.7	89.2	0.86	0.85	0.70	0.51
PSSM-SPINE-S	99.5	96.8	92.9	92.2	0.95	0.88	0.77	0.66

## Conclusion

In this study we proposed novel segmented distribution and segmented auto covariance feature extraction methods to capture more local and global discriminatory information from evolutionary profile and predicted secondary structure of proteins. We first extract the corresponding features from the PSSM in addition to the occurrence of the amino acids extracted from evolutionary consensus sequence and semi-composition extracted from the PSSM. Then by applying SVM to the extracted features, we enhanced the protein structural class prediction accuracy for low-homology protein sequences (twilight zone) up to 15.5% for the 25PDB benchmark and 5.1% for the 1189 benchmark better than similar studies that relied solely on the PSSM for feature extraction [26]. Our results supported the idea that potential sequence order information embedded in the PSSM has not been adequately explored in the literature.

In continuation, we added similar features extracted from the predicted secondary structure using the SPINE-X (segmented distribution, segmented auto covariance of the normalized probability of secondary structure elements, occurrence of secondary structure elements extracted from the structural consensus sequence, and semi-composition of the secondary structure elements extracted from the SPINE-M) to previously extracted features from the PSSM. By incorporating structural information, we achieved up to 92.2% and 86.3% for the 25PDB and the 1189 benchmarks which were respectively up to 7.9% and 2.8% better than previously reported results found in the literature for these two benchmarks that have been widely used for the protein structural class prediction problem [5, 6, 27].

## Future works

We are currently investigating the effectiveness of our proposed techniques in this study to tackle protein fold recognition. We are aiming to develop our protein structural class, and fold prediction server which will be publicly available in the near future. We also aim at exploring the-state-of-the-art feature reduction techniques on our extracted features to investigate the possibility of further feature reduction for these tasks.

## Declarations

Publication of this article funded by Griffith University and National ICT Australia (NICTA).

## Electronic supplementary material

Additional file 1: **Results as a function of k in k-fold cross validation The results achieved using SVM to the SPINE-S, PSSM-S, and PSSM-SPINE-S feature vectors using 2 to 10 fold cross validation for 25PDB and 1189 benchmarks**. (PDF 279 KB)
