# Supplementary material for: Proposing a highly accurate protein structural class predictor using segmentation-based features
Source: BMC Genomics. 2014 Jan 24;15(Suppl 1):S2. doi: 10.1186/1471-2164-15-S1-S2 (PMC4046757; doi:10.1186/1471-2164-15-S1-S2)

Appendix A: Results as a function of k in k-fold cross validation

The results achieved using SVM to the SPINE-S, PSSM-S, and PSSM-SPINE-S feature vectors using 2 to 10 fold cross validation for 25PDB benchmark

Table 1: The results (%) achieved for SPINE-S feature group using 2 to 10 fold cross validation evaluation method.

Cross validation	All - α	All - β	α / β	α + β	Overall
2 folds	93.7	82.4	72.9	71.2	80.4
3 folds	93.9	83.0	75.5	73.2	81.7
4 folds	93.9	83.0	76.4	73.0	81.8
5 folds	93.8	83.0	76.5	73.5	82.0
6 folds	94.0	83.0	76.8	73.2	82.0
7 folds	94.1	83.1	76.9	73.2	82.1
8 folds	93.8	83.2	77.2	74.0	82.1
9 folds	94.1	83.2	77.3	73.6	82.2
10 folds	93.9	83.3	77.2	73.1	82.2

Table 2: The results (%) achieved for PSSM-S feature group using 2 to 10 fold cross validation evaluation method.

Cross validation	All – α	All - β	α / β	α + β	Overall
2 folds	92.1	84.6	89.0	77.1	86.2
3 folds	93.2	85.4	90.1	78.6	87.3
4 folds	93.2	86.3	90.1	78.5	87.5
5 folds	93.4	87.0	90.6	79.2	88.0
6 folds	93.3	87.9	90.7	78.6	88.2
7 folds	93.5	87.9	90.5	79.9	88.4
8 folds	93.7	87.9	90.7	79.0	88.3
9 folds	93.9	87.9	90.4	79.3	88.3
10 folds	93.8	88.1	90.5	78.9	88.3

Table 3: The results (%) achieved for PSSM-SPINE-S feature group using 2 to 10 fold cross validation evaluation method.

Cross validation	All – α	All - β	α / β	α + β	Overall
2 folds	96.0	91.1	87.9	86.0	90.5
3 folds	96.1	91.6	89.7	87.6	91.4
4 folds	96.3	92.3	89.5	87.1	91.6
5 folds	96.3	92.3	90.0	87.3	91.7
6 folds	96.6	92.8	89.9	87.0	91.8
7 folds	96.6	92.8	89.7	86.7	91.7
8 folds	96.6	93.0	89.8	86.8	91.8
9 folds	96.5	92.9	89.8	87.3	91.8
10 folds	96.8	92.8	90.2	87.3	92.1

The results achieved using SVM to the SPINE-S, PSSM-S, and PSSM-SPINE-S feature vectors using 2 to 10 fold cross validation for 1189 benchmark

Table 4: The results (%) achieved for SPINE-S feature group using 2 to 10 fold cross validation evaluation method.

Cross validation	All - α	All - β	α / β	α + β	Overall
2 folds	90.3	87.2	76.0	56.7	77.6
3 folds	90.3	87.2	78.5	57.4	78.6
4 folds	90.7	87.0	78.4	57.6	78.6
5 folds	90.6	87.4	78.3	57.6	78.7
6 folds	90.9	87.3	78.1	58.7	78.8
7 folds	90.9	87.6	78.8	58.3	79.0
8 folds	91.3	87.8	78.4	57.4	78.9
9 folds	90.8	87.4	78.8	57.6	78.9
10 folds	91.2	87.6	79.1	57.8	79.0

Table 5: The results (%) achieved for PSSM-S feature group using 2 to 10 fold cross validation evaluation method.

Cross validation	All – α	All - β	α / β	α + β	Overall
2 folds	89.7	81.1	74.2	58.2	75.6
3 folds	91.3	84.5	75.1	62.8	78.2
4 folds	91.0	84.2	75.8	61.5	78.0
5 folds	91.5	84.8	77.2	65.0	79.4
6 folds	92.3	85.4	77.1	63.7	79.4
7 folds	91.7	85.3	78.0	64.2	79.5
8 folds	91.5	85.4	77.2	65.1	79.4
9 folds	92.4	85.7	77.1	64.9	79.5
10 folds	92.5	85.6	77.6	64.2	79.6

Table 6: The results (%) achieved for PSSM-SPINE-S feature group using 2 to 10 fold cross validation evaluation method.

Cross validation	All – α	All - β	α / β	α + β	Overall
2 folds	95.7	89.1	77.8	59.6	80.4
3 folds	96.8	91.0	78.9	63.7	82.4
4 folds	96.7	91.2	80.5	65.5	83.3
5 folds	96.8	91.4	81.5	66.1	83.7
6 folds	97.3	91.6	80.9	67.9	84.3
7 folds	97.1	92.2	82.0	67.2	84.4
8 folds	97.6	91.8	81.9	67.8	84.6
9 folds	97.5	92.0	82.4	68.7	85.0
10 folds	97.6	91.9	83.1	68.7	85.8

Figure 1: Comparison of the results achieved for PSSM-S, SPINE-S (for 25PDB benchmark), and PSSM-SPINE-S using 2 to 10 fold cross validation

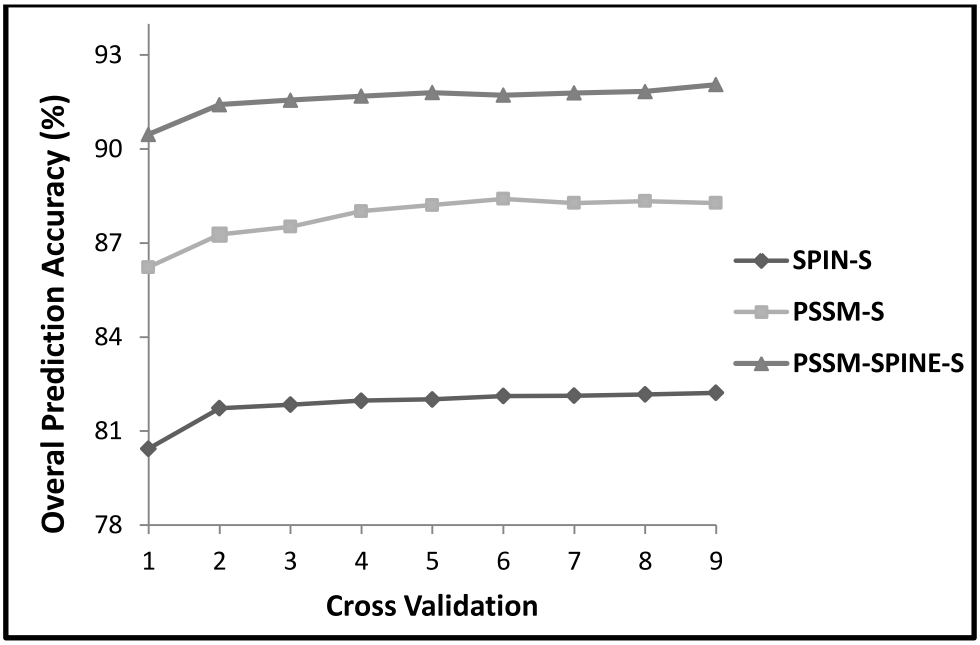

Figure 2: Comparison of the results achieved for PSSM-S, SPINE-S (for 1189 benchmark), and PSSM-SPINE-S using 2 to 10 fold cross validation

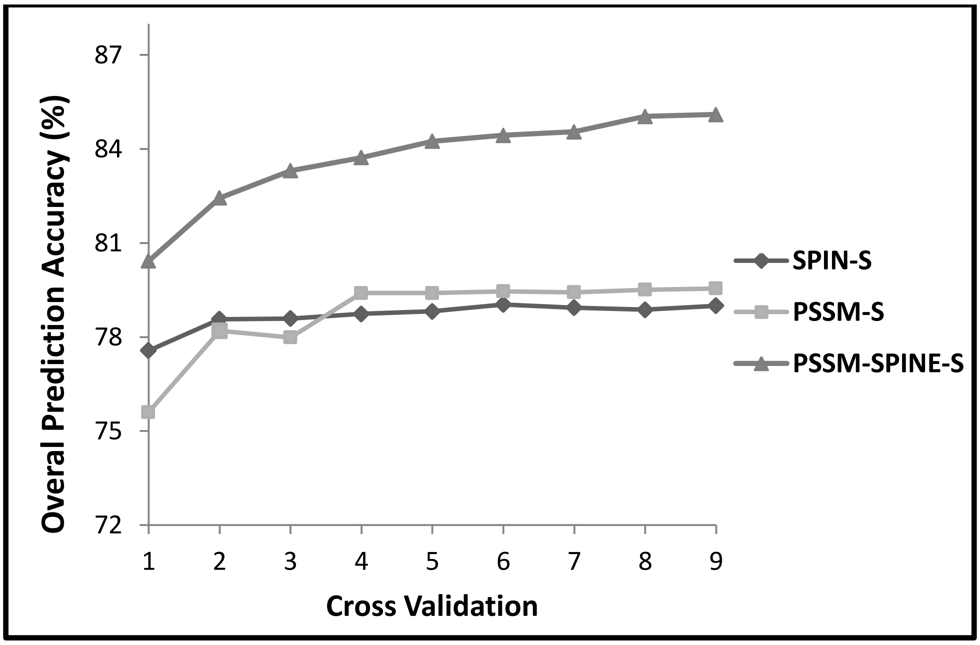

Supplement: Supplementary file 1 — Additional file 1: Results as a function of k in k-fold cross validation The results achieved using SVM to the SPINE-S, PSSM-S, and PSSM-SPINE-S feature vectors using 2 to 10 fold cross validation for 25PDB and 1189 benchmarks. (PDF 279 KB) [file 12864_2014_5675_MOESM1_ESM.pdf]
